# Nearly-octave wavelength tuning of a continuous wave fiber laser

**DOI:** 10.1038/srep42611

**Published:** 2017-02-15

**Authors:** Lei Zhang, Huawei Jiang, Xuezong Yang, Weiwei Pan, Shuzhen Cui, Yan Feng

**Affiliations:** 1 Shanghai Institute of Optics and Fine Mechanics, Chinese Academy of Sciences, and Shanghai Key Laboratory of Solid State Laser and Application, Shanghai 201800, China; 2 University of the Chinese Academy of Sciences, Beijing 100049, China

## Abstract

The wavelength tunability of conventional fiber lasers are limited by the bandwidth of gain spectrum and the tunability of feedback mechanism. Here a fiber laser which is continuously tunable from 1 to 1.9 μm is reported. It is a random distributed feedback Raman fiber laser, pumped by a tunable Yb doped fiber laser. The ultra-wide wavelength tunability is enabled by the unique property of random distributed feedback Raman fiber laser that both stimulated Raman scattering gain and Rayleigh scattering feedback are available at any wavelength. The dispersion property of the gain fiber is used to control the spectral purity of the laser output.

Widely wavelength tunable and spectrally bright fiber lasers are required in a variety of applications including spectroscopy, medicine, and scientific research^1^,^2^,^3^. Supercontinuum fiber sources can cover ultra-broad spectrum^4^,^5^,^6^, which however have limited spectral intensity. Traditional tunable fiber lasers explore the emission spectra of rare earth doped glass fibers, whose gain bandwidths are narrow with a maximum of about 200 nm^1^,^7^,^8^,^9^. Stimulated Raman scattering can provide optical gain at any wavelength in the fiber transparent window, if appropriate pump laser is present^10^,^11^,^12^,^13^. But the tuning range of conventional Raman fiber lasers was reported within 100 nm^14^,^15^, due to limited tunability of optical feedback mechanism necessary for laser action. Standard fiber lasers use narrow band fiber Bragg grating as cavity mirrors, or have a ring configuration with an intracavity filter for wavelength selection. The wavelength tuning capability of both fiber Bragg grating and intracavity filter is constrained. Raman soliton sources can be widely tunable. But it required femtosecond pulse laser as pump source and dispersion managed photonic crystal fiber as nonlinear medium. In addition, the output pulse energy is limited due to the soliton stability, and the spectral purity and spectral intensity of the achieved laser output is low as well^16^,^17^.

Random distributed feedback fiber laser offers a new possibility, since the feedback is provided by Rayleigh scattering, which is also available at any wavelength^18^,^19^. The wavelength tunability of random Raman fiber lasers was studied by cascaded Raman scattering, which can emit at discrete wavelengths^20^,^21^. Continuously wavelength tuning within a single Raman shift had achieved a less than 35 nm range^22^. Recently, by combining cascaded Raman scattering and tuning of pump laser wavelength, continuously wavelength tuning of 300 nm was reported^23^. Further wavelength extension resulted in reduced spectral purity and continuum spectrum.

Here, the mechanism which prevents the wavelength extension is clarified. Specialty silica fibers with zero dispersion wavelength longer than 2 μm are chosen as the gain medium of the random distributed feedback Raman fiber laser. With an improved laser setup, a fiber laser which can be continuously wavelength tuned from 1 to 1.9 μm is demonstrated. To the best of our knowledge, it is the widest wavelength tuning range ever reported for continuous wave fiber lasers.

## Results

**Figure 1:**
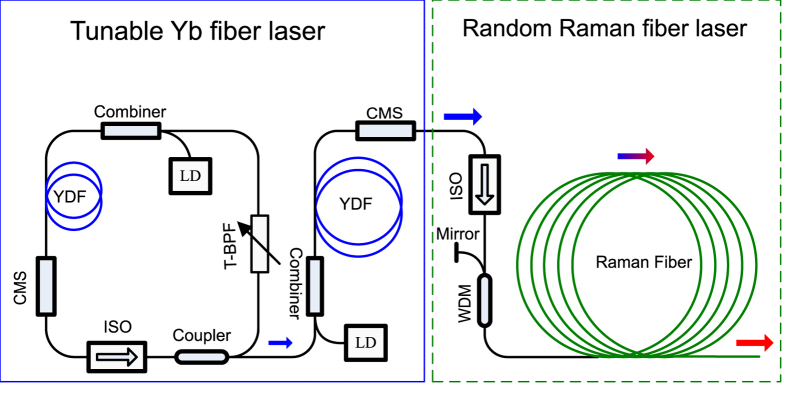
The configuration of the tunable fiber laser. It contains two main sections: a tunable Yb fiber laser as pump source and a random distributed feedback Raman fiber laser. YDF, Yb doped fiber; LD, laser diode; T-BPF, tunable bandpass filter; ISO, isolator; CMS, cladding mode stripper; WDM, wavelength division multiplexer.

The laser configuration is shown in Fig. 1. It includes a tunable Yb doped fiber master oscillator power amplifier as pump source, followed by a random distributed feedback Raman fiber laser. The output wavelength of the Yb doped fiber master oscillator can be tuned from 1000 to 1099 nm by the intracavity bandpass filter. After the Yb fiber power amplifier and isolator, more than 37.5 W can be obtained from 1020 to 1080 nm (Supplementary Information S1). The output from the tunable pump laser is optically isolated and injected into a 2000 m long piece of Raman fiber (OFS Raman optical fiber) after a wavelength division multiplexer. A broadband fiber pigtailed metallic mirror is attached to the rear free end of the wavelength division multiplexer, which forms a “half-open” random laser cavity together with the long piece of Raman fiber (Supplementary Information S2). The half-open configuration can greatly reduce the random laser threshold (Supplementary Information S3). The far end of Raman fiber is angle cleaved to minimize the back reflection. The randomly distributed Rayleigh scattering in the core of Raman fiber provides necessary feedback for the laser action.

**Figure 2:**
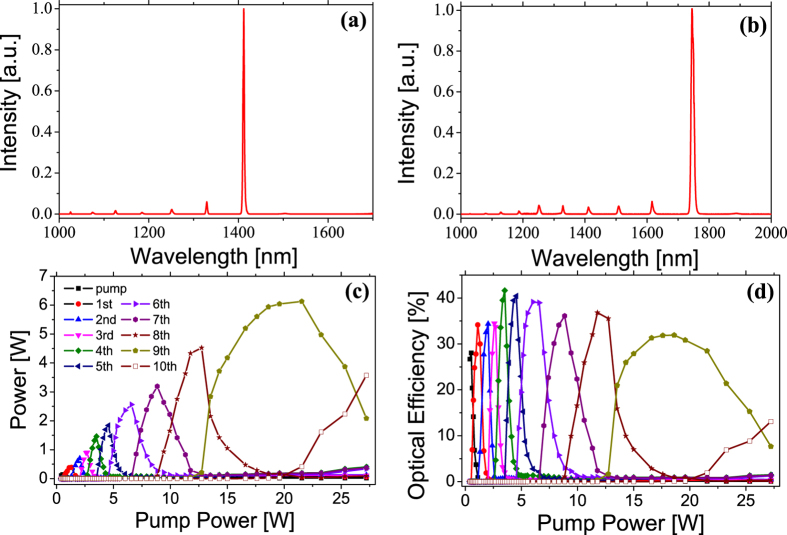
Cascaded Raman Stokes light generation with increasing pump power. The pump wavelength is 1025 nm in this case. (**a**) and (**b**) are the output spectra of the laser when optimized for the 6th and 9th order Raman Stokes. (**c**) and (**d**) are the output powers and efficiencies for different order Raman Stokes lights with respect to pump power.

The laser setup is examined first with fixed pump wavelength. At low pump laser power, broadband Raman amplified spontaneous emission is observed. With increasing pump power, the output spectrum narrows suddenly, showing a clear threshold behavior as one proof random lasing. Further increasing the pump power, Raman Stokes light can be generated cascadedly. In the case of 1025 nm pumping, up to 10th order Stokes light at 1.9 μm can be obtained (Supplementary Information S4). Output spectra of the laser optimized for the 6th and 9th Stokes light are shown in Fig. 2(a) and (b) as examples. Figure 2(c) and (d) summarize the output power and efficiency of different Raman Stokes light with respect to the pump power. The pump power is limited by allowed input of the optical isolator. The power and efficiency curves show obvious threshold behavior. A gradual power curve would be observed if the Raman Stokes light is generated by amplified spontaneous emission.

Notably, higher order Stokes light is generated almost successively, which means specific Stokes light can be selected just by tuning the pump power. In the experiments, more than 80% power purity can be readily achieved for up to 9th order Stokes light. This is a rather unique property of random Raman fiber laser among different cascaded Raman Stokes generation processes. In nested Raman oscillators and cascaded Raman fiber amplifiers, similar spectral purity can only be obtained within a narrow parameter range^12^,^24^. In spontaneous cascaded Raman amplification process pumped by a pulsed laser, supercontinuum-like multiple Raman Stokes output is usually obtained^25^.

The maximum conversion efficiency from pump to different Stokes orders ranges from 30 to 40%. A recent work on cascaded random lasing in polarization maintaining fiber reported about 2 times higher efficiency, where three Raman Stokes was achieved^26^. The lower efficiency here is due to the 2 times longer fiber used the experiments, which is necessary for achieving random lasing up to 10^th^ Raman Stokes. Another interesting observation is that the maximum optical efficiency is seen for the 4th Stokes light, even though more Raman shifting processes are required than the lower orders light. This is due to the loss spectrum of the germanium doped silica Raman fiber, which has a minimum around 1.5 μm, typically for all silica fibers. Because of the long fiber, the passive power loss predominates the quantum defect induced loss for the first 4 Raman Stokes.

When the pump wavelength varies, the wavelengths of the cascaded Raman Stokes light changes accordingly. The Raman shift is 440 cm^−1^ for germanium doped silica fiber. At 1 μm, it corresponds to a wavelength change of 46 nm. Therefore, if one adjusts the wavelength of a pump laser at 1 μm over 50 nm range, gapless tuning of cascaded Raman Stokes light can be realized.

**Figure 3:**
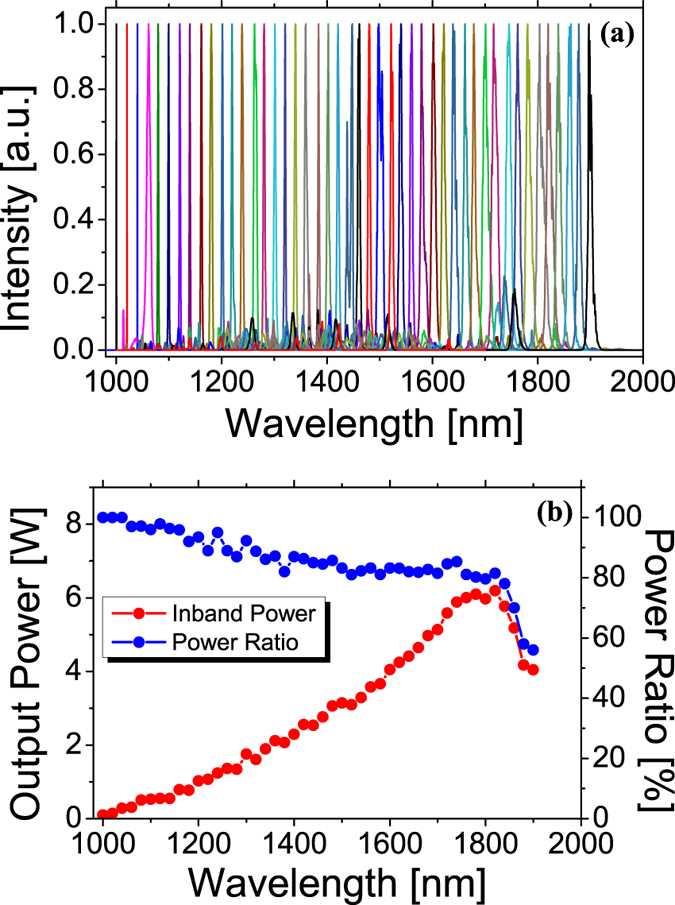
Continuously wavelength tuning from 1 to 1.9 μm. (**a**) Output spectra plotted for every 20 nm from 1 to 1.9 μm. (**b**) Output power and inband power ratio as a function of wavelength.

Figure 3 shows the result of continuously wavelength tuning from 1 to 1.9 μm. A time-lapse of the continuous wavelength tuning is shown in Supplementary Movie. For each output, the wavelength is determined by tuning the pump wavelength, and the spectral purity is optimized by adjusting the pump power. The output power increases with respect to the wavelength, because higher power is required to generate higher Stokes. The 3 dB laser linewidth increases from 2 nm to about 5 nm with increasing Stokes order and wavelength. And the spectral purity is higher than 80%. These behavior changes suddenly at about 1.8 μm, which is the 10th Stokes. The output power drops, and the conversion efficiency is low. The spectral purity decreases, and only 50% power ratio is achieved. This is due to the increased fiber loss at longer wavelength. The Raman fiber is specified to work at wavelength from 1.1 to 1.7 μm.

**Figure 4:**
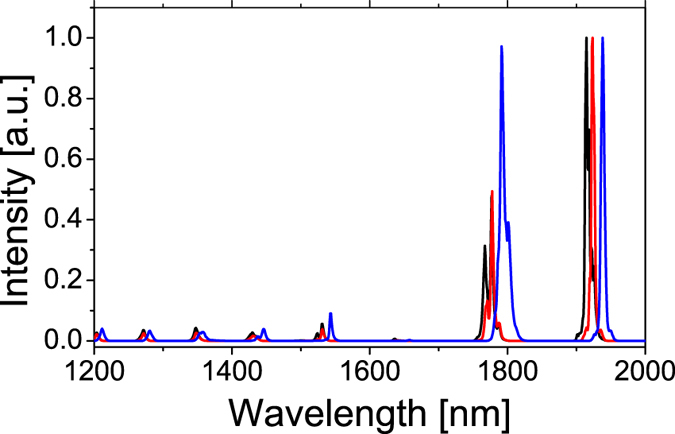
Further wavelength extension with ultra high NA fiber. Output spectra of laser generation at wavelength longer than 1.9 μm wavelength. As long as 1.94 μm is obtained with UHNA7 fiber.

To further extend into the longer wavelength, a 200 m long piece of UHNA7 fiber (Nufern inc.) is spliced after the Raman fiber. The UHNA7 fiber has a high germanium doping of ~58 wt.%, a core diameter of 2.4 μm and NA of 0.41, leading to a high Raman gain and low fiber loss at around 2 μm. As seen in Fig. 4, the wavelength is further extended to 1.94 μm. But the output is only 2.5 W due to the greater fiber loss. A further Raman shift into >2 μm regime would require higher injected pump power.

## Discussing

Note that the ultrawide wavelength tuning is enabled by choosing Raman fiber with zero dispersion wavelength >2 μm. The OFS Raman fiber have a zero dispersion wavelength of ~2.2 μm, and the UHNA7 fiber has an even longer zero dispersion wavelength of ~2.6 μm. As a comparison, SMF-28 fiber is also tested for random cascaded Raman scattering (Supplementary Information 5), whose zero dispersion wavelength is close to 1.3 μm. Successive Raman shifting beyond 1.3 μm is not possible, instead broadband supercontinuum-like light is generated due to the four wave mixing process, which is a common Kerr nonlinearity in optical fiber. Four wave mixing, which requires phase matching between the light waves, is usually a weak process. However, it becomes significant near zero dispersion wavelength, because of better phase matching condition. At this wavelength regime, with the assistance of four wave mixing, higher order Raman Stokes light can be generated at a relatively low threshold, which prevents the further power conversion from low order to high order Stokes laser.

In conclusion, we find that the random distributed feedback Raman fiber laser provides a route to achieve ultra-wide wavelength tunable output, because both stimulated Raman scattering gain and Rayleigh scattering feedback are available at any wavelength. Continuously wavelength tuning from 1 to 1.9 μm is achieved from a single laser system, by pump wavelength tuning and cascaded Raman shifting. Further extension of the wavelength range is possible by increasing the pump power and optimizing the gain fiber.

The described laser setup is a convenient tunable light source with off-the-shelf fiber components. In the experiments, the longest available Raman fiber in our lab is used to demonstrate the widest wavelength tuning. The fiber length is too long for shorter wavelength. Because the laser would be converted to longer wavelength at higher pump, the output power at shorter wavelength is low. For each narrower wavelength range, there is an optimum fiber length for maximum output. The laser source can find application in spectroscopy, scientific study, and biomedical research. One exciting prospect is to achieve rapid and ultrawide wavelength swept laser by rapid tuning of the pump laser, which could be an interesting source for optical coherence tomography imaging.

## Methods

The tunable Yb fiber pump laser has a standard master oscillator power amplifier configuration. Yb-doped polarization maintaining double-clad fibers with a core diameter of 10 μm and a numerical aperture of 0.075 are used as gain media for the seed laser and amplifier. They are pumped by 976 nm laser diodes, which have a nominal 4.8 dB/m absorption in the gain fiber. The seed laser has a ring cavity geometry. A tunable bandpass filter based on the thin film cavity is used to select the operating laser wavelength, which is tunable from 1000 to 1099 nm with a tuning resolution of 0.02 nm and a full width half maximum bandwidth of 1 nm. The amplifier is built and optimized for the 1020–1080 nm wavelength range. The whole pump laser system is all-fibered and polarization-maintained.

Then the tunable pump laser is injected into the Raman random laser through a broadband (1025–1080 nm) isolator to prevent the backward distributed Rayleigh feedback into the amplifier, which would cause parasitic lasing and may destroy the amplifier. A wavelength division multiplexer is spliced between the isolator and the Raman gain fiber. A fiber pigtailed broadband reflection mirror is spliced with the free port at the input side of the pump laser to construct the half-open random fiber laser. A piece of 2 km-long Raman fiber (from OFS Optics) is used as the gain fiber which supplies the distributed Rayleigh scattering and the Raman gain simultaneously. The output end of this fiber is cleaved at an angle >8° to suppress the backward reflection.

The laser tuning range from 1 to 1.94 μm cannot be covered by a single optical spectrum analyzer. Therefore, two optical spectrum analyzers, Yokogawa AQ6370D and AQ6470B, are used to measure the output spectra from 1000–1700 nm and 1200–2000 nm, respectively.

## Additional Information

**How to cite this article:** Zhang, L. *et al*. Nearly-octave wavelength tuning of a continuous wave fiber laser. *Sci. Rep.* **7**, 42611; doi: 10.1038/srep42611 (2017).

**Publisher's note:** Springer Nature remains neutral with regard to jurisdictional claims in published maps and institutional affiliations.

## Supplementary Material

Supplementary Information

Supplementary Video
