# Supplementary material for: Nearly-octave wavelength tuning of a continuous wave fiber laser
Source: Sci Rep. 2017 Feb 15;7:42611. doi: 10.1038/srep42611 (PMC5309793; doi:10.1038/srep42611)
Supplement: Supplementary Information [file srep42611-s1.pdf]

Nearly-octave wavelength tuning of a continuous wave fiber laser

Lei Zhang,[1] Huawei Jiang,[1,2] Xuezong Yang,[1,2] Weiwei Pan,[1,2] Shuzhen Cui,[1] and Yan Feng[1,*]

[1]Shanghai Institute of Optics and Fine Mechanics, Chinese Academy of Sciences, and Shanghai Key Laboratory of Solid State Laser and Application, Shanghai 201800, China
[2]University of the Chinese Academy of Sciences, Beijing 100049, China
*corresponding author: feng@siom.ac.cn

Section 1. The output performance of the tunable Yb fiber laser

The wavelength tunable pump laser is optimized to lase from 1020-1080 nm. As is depicted in Fig. 1, the output power after the isolator is over 37.5 W from 1020 to 1080 nm which is used to pump the random fiber laser. The maximum output power reaches 40.2 W at 1045 nm, which is limited by the power handling ability of the isolator. The amplified spontaneous emission (ASE) is 50 dB lower than the laser line in the emission band.

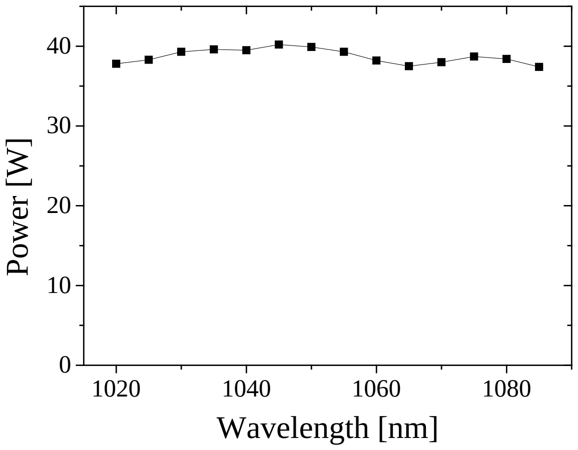

Fig.1 Output power of the tunable pump laser from 1020 to 1080 nm.

Section 2. The spectral property of the wavelength division multiplexer and the broadband metallic mirror

The transmission spectra of the WDM are shown in Fig. 2a, measured with a supercontinuum laser source (600-2000nm). The red and black curves correspond to the transmission from the pump port to output port and mirror port to the output port, respectively. At the pump laser band from 1020 to 1075nm, the insertion loss is well below 1.2dB. At the tunable random Raman laser band from

1060 to 1940nm, the transmission spectrum varies up and down. Figure 2b gives the reflectivity spectrum of the gold mirror (Thorlabs, PF10-03-M01), which have a reflectivity higher than 96 % from 1 to 2 μm. The mirror is packaged with SMF-28 fiber pigtail.

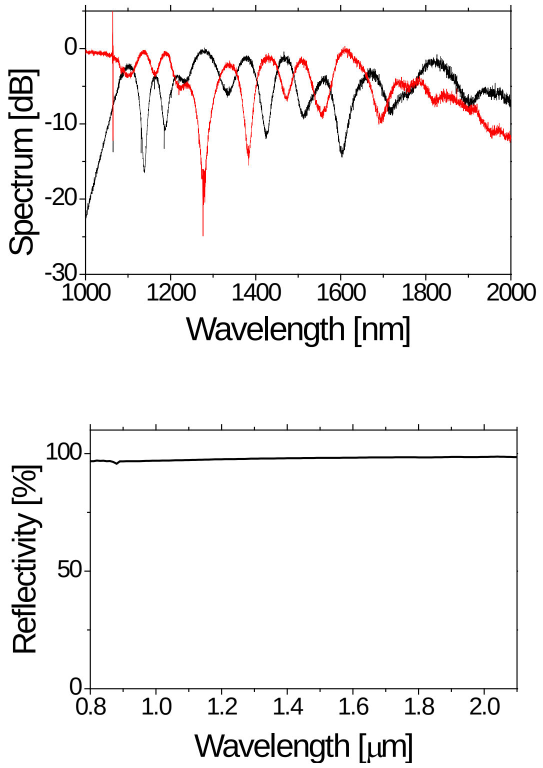

Fig.2 a) The transmission property of the WDM. b) the reflectivity spectrum of the metallic mirror.

Section 3. Lower laser threshold with "half-open" random cavity.

The "half-open" cavity decrease the random laser threshold greatly. The effectiveness of the broadband mirror is examined by comparing the laser output with and without the mirror. Figure 3 shows the random laser output spectra pumped by a 35 W 1062 nm laser, when the rear end is finished with the mirror, a zero degree cleave, and a 10 degree angled cleave, respectively. The zero degree cleave provides a reflectivity of about 4% due to the Fresnel reflection, while the angled cleave suppresses the reflection from the fiber end. It is seen that with the HR mirror up to 9th cascaded random Raman laser is generated, while with the zero degree and angled cleaves only up to 7th and 6th order random Raman laser is achieved, respectively.

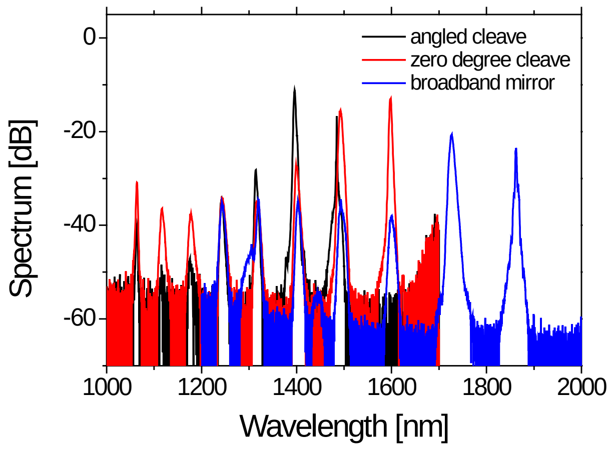

Fig.3 The laser output spectra at different rear fiber end conditions.

Section 4. Cascaded generation of high order Raman Stokes light

With a fixed pump wavelength at 1025nm, the typical cascaded Raman random laser output spectra optimized for each Raman Stokes are shown in Fig. 4. With the increase of pump laser power, 1st to 10th order Raman emissions are generated successively.

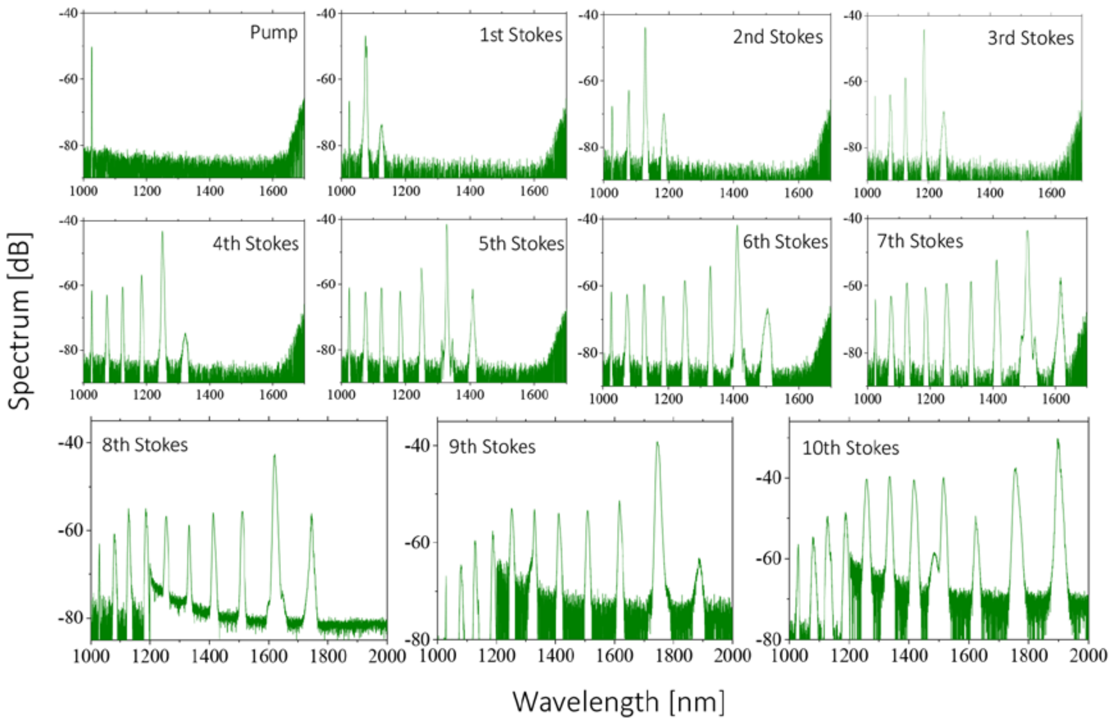

Fig. 4 Typical output spectra of the random laser for the cases of pump only and 1st to 10th order Stokes light.

Section 5. Cascaded random distributed feedback Raman fiber laser with SMF 28 fiber.

The Raman fiber used in the experiment is replaced by a piece of 12 km long SMF-28 fiber for comparison. The output spectrum with a 10.2 W 1070 nm laser pump is shown in Fig. 5. Only three narrowband Raman Stokes light can be generated. The 4th Stokes light is broadband.

The zero dispersion wavelength of SMF-28 fiber is around 1310 nm. Near the zero dispersion wavelength, the four wave mixing (FWM) process is efficient. The FWM between the Raman Stokes lights results in the low threshold generation of the higher order Raman emission. In addition, the 4th order Raman light is spectrally broadened due to nonlinear processes, which prohibits the further wavelength expanding of the random Raman fiber laser.

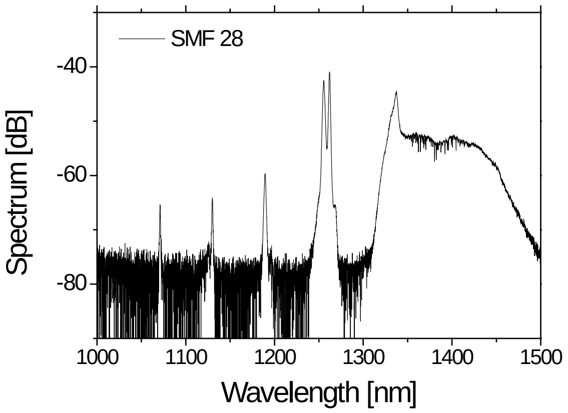

Fig.5 Typical output spectrum with SMF-28 fiber and Raman gain medium.

Movie

A time-lapse of the continuous wavelength tuning. For each output, the wavelength is determined by tuning the pump wavelength, and the spectral purity is optimized by adjusting the pump power.
